# Heat-Related Deaths After an Extreme Heat Event — Four States, 2012, and United States, 1999–2009

**Published:** 2013-06-07

**Authors:** David R. Fowler, Clifford S. Mitchell, Alise Brown, Tessie Pollock, Lynne A. Bratka, John Paulson, Anna C. Noller, Robert Mauskapf, Kathryn Oscanyan, Ambarish Vaidyanathan, Amy Wolkin, Ethel V. Taylor, Rachel Radcliffe

**Affiliations:** Maryland Office of the Chief Medical Examiner; Maryland Dept of Health and Mental Hygiene; Ohio Dept of Health; Virginia Office of the Chief Medical Examiner; Virginia Dept of Health; West Virginia Dept of Health and Human Resources; Div of Environmental Hazards and Health Effects, National Center for Environmental Health; Career Epidemiology Field Officer Program, CDC

On June 29, 2012, a rapidly moving line of intense thunderstorms with high winds swept across the midwestern and eastern United States, causing widespread damage and power outages. Afterward, the area experienced extreme heat, with maximum temperatures exceeding 100°F (37.8°C) (1). This report describes 32 heat-related deaths in Maryland, Ohio, Virginia, and West Virginia that occurred during the 2 weeks following the storms and power outages. Median age of the decedents was 65 years, and most of the excessive heat exposures occurred within homes. During 1999–2009, an annual average of 658 heat-related deaths occurred in the United States (2). Heat-related deaths are preventable, and heat response plans should be in place before an extreme heat event (EHE). Interventions should focus on identifying and limiting heat exposure among vulnerable populations.

During June 30–July 13, 2012, an EHE occurred; maximum daily temperatures in Maryland, Ohio, Virginia, and West Virginia ranged from 83°F to 104°F (28.3°C to 40.0°C), averaging 9.5°F (5.3°C) warmer than normal (1). The EHE followed a series of powerful thunderstorms with wind gusts up to 80 miles (129 km) per hour that caused widespread damage across parts of the Ohio Valley and the Mid-Atlantic regions. The resultant power outages affected approximately 3.8 million persons and lasted 8 days in some areas. To describe the epidemiology of heat-related deaths that occurred during the EHE, information was collected from the state offices of the medical examiner or vital statistics. These offices analyzed death certificates and medical examiners’ records and recorded deaths in which exposure to excessive heat either caused or significantly contributed to a death.* For comparison, a baseline number of heat-related deaths† in these four states over the same 2-week summer period each year of 1999–2009 was calculated using mortality data from CDC (2).

During June 30–July 13, 2012, a total of 32 deaths (0.11 deaths per 100,000 population) from excessive heat exposure were reported, including 12 in Maryland, 12 in Virginia, seven in Ohio, and one in West Virginia. In comparison, a median of four and average of eight (range: 1–29) heat-related deaths occurred in the four states during the same 2-week summer period each year of 1999–2009. The median age of the 32 decedents was 65 years (range: 28–89 years); 72% were male. Most decedents (75%) were unmarried or living alone. Common underlying or contributing conditions included cardiovascular disease (14) and chronic respiratory disease (four). In at least seven (22%) of the deaths, loss of power from the storms was known to be a contributing factor. Overall, 22 (69%) decedents died at home, with lack of air conditioning reported in 20 (91%) of these deaths. In the homes of five persons who died, a functioning air conditioner was present but not turned on. Of the seven deaths in which housing type was specified, six occurred in multifamily dwellings. Heat exposure occurred outdoors in three deaths, and two deaths occurred in a vehicle.

To compare the 2012 EHE with previously reported EHEs without concurrent power outages, a search was conducted using PubMed for reports of deaths from EHEs that occurred in the United States during the previous 20-year period. The search was conducted using the key words “heat wave,” “extreme heat,” or “extreme heat event” plus the key words “mortality” or “death.” Only reports that covered a similar length of time (10–14 days) were included; a total of three reports met these criteria. During July 6–16, 1993, an EHE in Philadelphia, Pennsylvania, resulted in 118 deaths (7.5 deaths per 100,000) (3). Two years later, 514 deaths (9.7 deaths per 100,000) occurred during July 10–20, 1995, in Chicago, Illinois (4). In 2005, a 14-day heat wave resulted in 28 reported deaths (0.77 deaths per 100,000) in Maricopa County, Arizona (5). A lower fatality rate for heat-related deaths was reported in the 2012 EHE than in previous EHEs lasting 10–14 days. Public health and emergency management officials in Maryland, Ohio, Virginia, and West Virginia rapidly initiated preplanned heat response activities, which might have led to a decrease in the number of expected deaths.

To better understand the scope of heat exposure, mortality data for 1999–2009 (2) were used to review heat-related deaths in the United States overall. During this period, 7,233 heat-related deaths occurred, an average of 658 per year (Figure). In 5,201 (72%) of these deaths, the underlying cause was exposure to excessive heat, and heat was a contributing factor in the remaining 2,032 (28%) deaths. Heat-related deaths were reported most frequently among males (4,955; 69%) and among adults aged ≥65 years (2,621; 36%). Almost all heat-related deaths occurred during May–September (6,821; 94%), with the highest numbers reported during July (2,825; 39%) and August (1,925; 27%).§

## Editorial Note

EHEs, defined as summer temperatures substantially hotter or more humid than the norm for the location and time of year, lead to increased numbers of heat-related illnesses and deaths. The number of heat-related deaths reported during the 2012 EHE, which coincided with widespread power outages, was higher than the average number of heat-related deaths reported in these four states in the same 2-week period for previous years. Of the 32 persons who died, half were aged ≥65 years. Based on medical examiner reports, lack of air conditioning and the type of housing contributed to some of these deaths.

Heat-related deaths are preventable, and advanced planning for EHEs is recommended to minimize mortality during these events (6,7). Identifying vulnerable populations (e.g., the elderly, very young persons, persons with chronic illnesses, or those with altered mental status) and targeting interventions to those most at risk are keys to prevention. Interventions during an EHE include staying cool, hydrated, and informed about extreme heat alerts in the area and symptoms of heat illness.

Several states developed interventions targeting the elderly during the 2012 EHE. In Ohio, the Emergency Management Agency, the Department of Health, and the Department of Aging collaborated to identify areas of high concentrations of power outages and high populations of older residents. Beginning July 1, approximately 200 National Guard personnel conducted home visits to the elderly to identify persons experiencing signs of heat exhaustion using wellness toolkits prepared by these three organizations. On July 2, Ohio launched a “Check on Your Neighbor” campaign to encourage residents to help identify and assist persons at risk. On July 3, the Ohio Board of Regents and Department of Aging enlisted the aid of university and college students as part of the “Knock and Talk” effort targeting the elderly. The National Guard in West Virginia also participated in home visits to the elderly and other socially isolated persons, with approximately 100 health and wellness teams going door-to-door in communities throughout the state. In Maryland, assisted-living programs servicing ≥50 persons are required to have an emergency electrical power generator onsite.¶

Utility companies in Virginia and West Virginia were represented in the emergency operations centers from the onset of the EHE and worked with the states to prioritize power restoration to vulnerable populations. States also used multiple media formats (e.g., press releases, media interviews, social media, reverse 911 calls, and daily web updates) to communicate rapidly and frequently with the public and provide educational messages and increased awareness of resources.

In Virginia, pre-scripted public information messages about the dangers of excessive heat exposure and available resources are prepared before summer begins. Developing communication plans before an event allows for a quicker response (8), and enables staff to focus on other intervention activities.**

Municipalities can develop heat response plans in preparation for EHEs. In 2011, the Maryland Department of Health and Mental Hygiene developed a heat emergency plan that outlines actions to be taken before the beginning of the extreme heat season and provides guidance during an EHE. Under this plan, educational messages regarding heat exposure risks are issued beginning in June. Although the combination of widespread power outages and high temperatures was unexpected, public awareness in Maryland of the risks associated with excessive heat exposure likely was heightened as a result of educational messages.††

In the 2012 EHE, 69% of decedents were found at home without air conditioning. Five decedents had an air conditioner that was not turned on. Power loss might have contributed to these deaths; decedents might have been unaware that power had been restored before they succumbed to heat. To increase access to air conditioning, cooling stations or other public locations could be opened to provide residents temporary relief from heat, particularly when elevated temperatures occur for several consecutive days. However, qualitative studies suggest that cooling stations are not well-used because of perceived and real barriers, including lack of transportation access, safety issues, stigma of public shelters, inability to bring pets, and limited operating hours (Sabrina McCormick, PhD, George Washington University, personal communication, 2013).

What is already known on this topic?

Excessive heat is a leading cause of preventable, weather-related deaths, particularly among the elderly. Recommended interventions for individuals include staying cool, hydrated, and informed.

What is added by this report?

During June 30–July 13, 2012, a total of 32 persons died from excessive heat exposure in four states. Their median age was 65 years (range: 28–89 years); 72% were male, and 75% were unmarried or living alone. Overall, 22 (69%) decedents died at home, with lack of air conditioning reported in 20 (91%) of these deaths. Despite widespread power outages, the numbers of heat-related deaths were lower than expected compared with the numbers occurring in previous extreme heat events.

What are the implications for public health practice?

Although evaluating the efficacy of heat response plans is difficult, advanced planning for extreme heat events and rapid, coordinated responses among state and local agencies and public and private entities might minimize the loss of life during a heat event and should be encouraged.

The findings in this report are subject to at least four limitations. First, because only deaths in which excessive heat exposure was recorded on the death certificate were reported, the number of deaths in which heat was a contributing factor might be underestimated (7). Second, although the 14-day reporting period was chosen on the basis of surveillance data, maximum daily temperatures, and time to power restoration, some deaths caused by this event might have occurred after July 13. Third, because historical numbers were based on codes assigned by the National Vital Statistics System (NVSS) and deaths reported in the 2012 EHE were based on death certificates, discrepancies might have occurred in how deaths were classified. Finally, because a few heat-related deaths occur each year in these four states, some of the deaths captured might have been part of the background rate and not a result of loss of power during the EHE. The number of deaths that might have occurred in these states regardless of the 2012 EHE could not be quantified because the historical numbers varied from year to year.

The targeted interventions for vulnerable populations that were implemented by the affected states might have reduced the loss of life from this EHE. Interventions, including rapid distribution of public health messages (e.g., reverse 911 calls), visits to persons at high risk, and laws to provide additional resources, (e.g., back-up power supplies to vulnerable populations), might have contributed to lower numbers of heat-related deaths. Public health and emergency management personnel should work together to identify vulnerable populations in their area and design response plans to guide actions during an EHE.

## Figures and Tables

**FIGURE:**
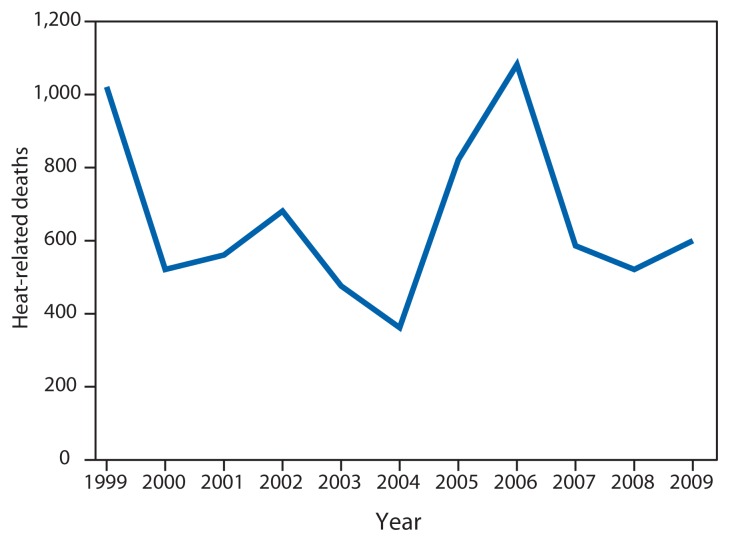
Heat-related deaths — United States, 1999–2009 **Source:** National Vital Statistics System. Mortality public use data files, 1999–2009. Available at http://www.cdc.gov/nchs/data_access/vitalstatsonline.htm.

## Footnotes

* The underlying cause of death was defined as the disease or injury that initiated the chain of events that led directly and inevitably to death. Contributing conditions, or factors, were defined as diseases, injuries, or complications that contributed to the death and were a result of the underlying cause. A sample death certificate, showing underlying and contributing causes of death, is available at http://www.cdc.gov/nchs/data/dvs/death11-03final-acc.pdf.

† Deaths from excessive heat exposure were defined using codes from the *International Classification of Diseases, 10th Revision*. Such deaths included those in which exposure to excessive natural heat (X30) was reported as either the underlying or a contributing cause of death. Deaths from exposure to excessive heat of man-made origin (W92) were excluded. Guidance for certification of death is available in the *Medical Examiners’ and Coroners’ Handbook on Death Registration and Fetal Death Reporting* (2003 revision), available at http://www.cdc.gov/nchs/data/misc/hb_me.pdf.

§ Additional analyses are available at www.ephtracking.cdc.gov/showhome.action.

¶ Emergency electrical power generator. Maryland Code Health–General, Title 19, Subtitle 18, Sect. 19-1812 (2010).

** A media toolkit for extreme heat, including resources targeted at specific groups, is available at http://www.cdc.gov/nceh/extremeheat.

†† Sample educational materials are available at http://www.cdc.gov/nceh/extremeheat/materials.html.

## References

[1] National Oceanic and Atmospheric Administration (2012). NOWData.

[2] Kochanek K, Xu J, Murphy S, Minino A, Kung H (2011). Deaths: final data for 2009. Natl Vital Stat Rep.

[3] Mirchandani HG, McDonald G, Hood I, Fonseca C (1996). Heat-related deaths in Philadelphia—1993. Am J Forensic Med Pathol.

[4] Whitman S, Good G, Donoghue ER, Benbow N, Shou W, Mou S (1997). Mortality in Chicago attributed to the July 1995 heat wave. Am J Public Health.

[5] Yip FY, Flanders WD, Wolkin A (2008). The impact of excess heat events in Maricopa County, Arizona: 2000–2005. Int J Biometeorol.

[6] World Health Organization (2008). Heat-health action plans guidance.

[7] US Environmental Protection Agency (2006). Excessive heat events guidebook.

[8] US Department of Homeland Security (2008). National response framework.

